# Running and Swimming Differently Adapt the BDNF/TrkB Pathway to a Slow Molecular Pattern at the NMJ

**DOI:** 10.3390/ijms22094577

**Published:** 2021-04-27

**Authors:** Laia Just-Borràs, Víctor Cilleros-Mañé, Erica Hurtado, Olivier Biondi, Frédéric Charbonnier, Marta Tomàs, Neus Garcia, Josep Tomàs, Maria A. Lanuza

**Affiliations:** 1 Unitat d’Histologia i Neurobiologia (UHNEUROB), Facultat de Medicina i Ciències de la Salut, Universitat Rovira i Virgili, 43201 Reus, Spain; laia.just@urv.cat (L.J.-B.); victor.cilleros@estudiants.urv.cat (V.C.-M.); erica.hurtado@urv.cat (E.H.); marta.tomas@urv.cat (M.T.); mariadelesneus.garcia@urv.cat (N.G.); 2 INSERM UMRS 1124, Université de Paris, CEDEX 06, F-75270 Paris, France; olivier.biondi@parisdescartes.fr (O.B.); frederic.charbonnier@u-paris.fr (F.C.)

**Keywords:** BDNF/TrkB signaling, endurance exercise, neuromuscular junction, skeletal muscle, new activity conditions

## Abstract

Physical exercise improves motor control and related cognitive abilities and reinforces neuroprotective mechanisms in the nervous system. As peripheral nerves interact with skeletal muscles at the neuromuscular junction, modifications of this bidirectional communication by physical activity are positive to preserve this synapse as it increases quantal content and resistance to fatigue, acetylcholine receptors expansion, and myocytes’ fast-to-slow functional transition. Here, we provide the intermediate step between physical activity and functional and morphological changes by analyzing the molecular adaptations in the skeletal muscle of the full BDNF/TrkB downstream signaling pathway, directly involved in acetylcholine release and synapse maintenance. After 45 days of training at different intensities, the BDNF/TrkB molecular phenotype of trained muscles from male B6SJLF1/J mice undergo a fast-to-slow transition without affecting motor neuron size. We provide further knowledge to understand how exercise induces muscle molecular adaptations towards a slower phenotype, resistant to prolonged trains of stimulation or activity that can be useful as therapeutic tools.

## 1. Introduction

Motor neurons (MNs) interact with skeletal muscles through the neuromuscular junction (NMJ) to regulate themselves in a feedback loop mode [1,2,3]. Thus, through the neuromotor and neurotrophic control, they guarantee an appropriated formation, maintenance, and functionality of each other. Therefore, circumstances that modify this bidirectional communication, such as an increase or decrease in physical activity, may result in positive or pathological adaptations, respectively [4,5,6].

Exercise is the most common physiological stimulus in humans and animals and can modify tissue functionality. Through the years, the benefits of physical activity have been functionally and morphologically studied in both nerves and muscles [6]. In particular, exercise is beneficial for the nervous system [7] as it improves motor control and cognitive abilities and reinforces neuroprotective mechanisms in brain [8], spinal cord [9,10], peripheral nerves, and muscles [11]. Among others, it induces cellular adaptations [12,13], increases mitochondrial capacity [14], and raises neurotrophic factor (NTF) expression [9].

Endurance and resistance are the two major categories of exercise normally distinguished. While resistance, characterized by short periods of activity at maximal intensity, like weightlifting, is slightly studied in small animals like rodents, endurance is extensively examined. It is characterized by extended periods of continuous neuromuscular activity at moderate, submaximal intensity and is equivalent to running, swimming, and bicycling. Endurance refines the NMJ function [15,16,17,18] and the morphology of the pre- and postsynaptic elements [19,20,21,22,23,24,25,26,27], resulting in a better performance of the neuromuscular system. After endurance, miniature and evoked end-plate potential (MEPP and EPP) amplitude, quantal content, and safety margin increase while MEPP frequency decreases without affecting the resting membrane potential. Furthermore, the number of synaptic vesicles and the degree of postsynaptic depolarization required to open voltage gated postsynaptic ion channels is modified [15,18]. This increase of neuromuscular activity is accompanied by a fast-to-slow transition in the target muscles while a lack of activity does the opposite [28]. Altogether, changes in the activity pattern of muscles result in reversible adaptations to new situations [29].

Fast-twitch muscles undergo more evident adaptations than slower muscles [30,31]. However, the involved molecular pathways are still poorly understood. We have recently found that the Brain-derived neurotrophic (BDNF)/Tropomyosin receptor kinase B (TrkB) signaling pathway, including kinases directly involved in the acetylcholine (ACh) release machinery, are differently expressed in slow and fast muscles. Indeed, this differentiation allowed to demonstrate that the amyotrophic lateral sclerosis (ALS)-induced fast-to-slow transition could also be observed at the molecular level in relation with neurotrophic factors misbalance [32].

The BDNF/TrkB signaling is one of the most implicated in the NMJ stability and is essential for neurotransmission [3,33,34,35,36,37]. BDNF is strongly expressed in skeletal muscle in response to muscle contraction [3,38]. Each BDNF isoform binds distinct receptors to mediate divergent neuronal actions [33,39,40,41]. ProBDNF preferentially interact with p75 neurotrophic factor receptor (p75^NTR^), whereas mature NTF forms, mBDNF, and neurotrophin-4 (NT4) selectively bind and activate the specific TrkB receptor [33,39,40,41]. Alternative splicing generates one TrkB full length isoform (TrkB.FL), which has an intracellular kinase domain, and two truncated isoforms (TrkB.T1 and TrkB.T2) that lack of it [42,43]. TrkB.FL, when it is not inhibited by TrkB.T1 heterodimerization [44,45,46], activates presynaptic protein kinases C (PKC) once they are phosphorylated by phosphoinositide-dependent kinase 1 (PDK1) [47], that modulate ACh release at the NMJ [3,48,49] by phosphorylating proteins of the exocytotic machinery such as Munc18-1 and synaptosomal nerve-associated protein 25 (SNAP-25) [50,51]. Furthermore, SNAP-25 is also phosphorylated by the cAMP-dependent protein kinase A (PKA) whose activity depends on muscarinic ACh receptors [52], which regulate the activation of TrkB [37,53]. Therefore, the optimization of this signaling pathway with exercise could strongly improve the functionality of NMJ. In accordance, the recovery of the BDNF/TrkB signaling with physical exercise in an ALS mice model was beneficial as it reduced MN loss [54].

Here we provide a relevant step between the external action of physical activity (whose effects are based in variations in protein transcription and translation), and the molecular reaction of the neuromuscular system by analyzing the full BDNF/TrkB signaling pathway, that works presinaptically to control synaptic vesicles exocytosis, including two classic and one novel downstream PKC isoforms (cPKCβI, cPKCα, and nPKCε), regulatory and catalytic PKA subunits and two components of the SNARE/SM exocytotic machinery proteins (Munc18-1 and SNAP-25). Thus, this study elucidates fast plantaris muscle’s ability to adapt to exercise, either running or swimming, resulting in a fast-to-slow molecular transition. The results show that molecular changes in the BDNF/TrkB pathway are essential for the plastic and synaptic-protective effect of exercise.

## 2. Results

We analyzed total and phosphorylated protein levels of the BDNF/TrkB downstream signaling pathway including BDNF and NT4 NTFs; their receptors TrkB and p75^NTR^; three downstream PKCs (α, βI and ε) and its priming kinase (PDK1); the different PKA subunits; and two PKC and PKA targets related with neurotransmitter release (Munc18-1 and SNAP-25).

Table 1 includes a summary of the changes induced by both trainings. Furthermore, the first data column shows the values of untrained soleus extracted from [32] to show the molecular similitudes between fast trained muscles and untrained slow ones to help the interpretation of the molecular adaptations.

### 2.1. Neurotrophic Factors and Receptors

**Figure 1:**
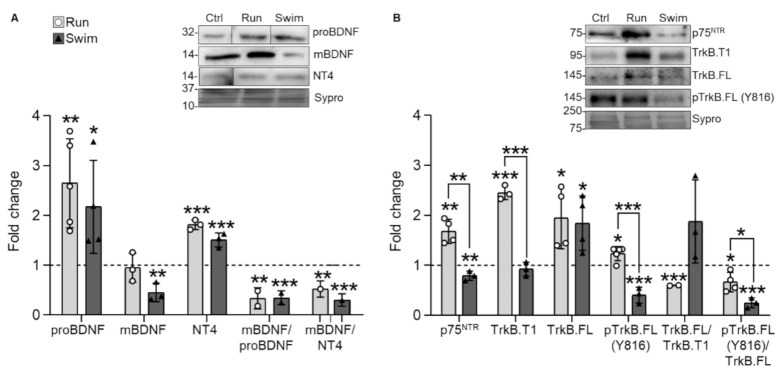
NTFs and receptors expression changes in trained plantaris muscles of WT mice. (**A**) Running and swimming increase proBDNF and NT4 levels while swimming decreases mBDNF. (**B**) Running increases all p75^NTR^, TrkB.T1, TrkB.FL, and pTrkB.FL while swimming decreases p75^NTR^ and pTrkB.FL and increases TrkB.FL. Data are presented as mean ± SD. Each dot in the bars is the mean result of one animal. * *p* ≤ 0.05; ** *p* ≤ 0.01; *** *p* ≤ 0.001; (Kruskal–Wallis test and Dunn’s post hoc test).

Results show that proBDNF is significantly increased by both training protocols compared with untrained animals. However, mBDNF protein levels are not affected by running but significantly decrease after swimming. Despite this difference in mBDNF, the mBDNF/proBDNF ratio decreases similarly and significantly after both protocols (Figure 1A). This may indicate that exercise increase precursor NTFs production of but that each training protocol has different consumption requirements. Thus, the utilization and subsequent degradation of mBDNF depends on the protocol. Furthermore, mature NT4 significantly increases after both protocols, which results in a decrease in the mBDNF/NT4 ratio (Figure 1A). Thus, both running and swimming have a similar effect over the prevalence of proBDNF and NT4 despite that the downstream signaling may be different due to distinct affinity of mBDNF for binding to TrkB and degradation. Interestingly, NT4 accumulation and mBDNF decrease are typical features of the slow soleus muscle as compared with the fast plantaris while proBDNF accumulation does not follow the fast-to-slow tendency (see Table 1).

As physical exercise modulates NTF levels, we next analyzed the fundamental receptors p75^NTR^ and TrkB (TrkB.T1, TrkB.FL and phosphorylated TrkB.FL -pTrkB.FL (Y816)-). Running significantly increases all four receptors, but especially TrkB.T1, which results in TrkB.FL/TrkB.T1 and, consequently, pTrkB.FL/TrkB.FL ratios being significantly decreased (Figure 1B), as a result of TrkB.T1 repression of the TrkB.FL phosphorylation. By the contrary, swimming decreases p75^NTR^ and pTrkB.FL while increases TrkB.FL and does not modify TrkB.T1. Thus, while the TrkB.FL/TrkB.T1 ratio increases, the pTrkB.FL/TrkB.FL ratio significantly decreases in swimming (Figure 1B). Altogether, each training protocol influences the NTF receptor changes differently, being the difference of expression between the dominant negative TrkB.T1 and the active TrkB.FL phosphorylation the more conspicuous. In particular, TrkB.FL dominance over TrkB.T1 could explain the mBDNF and pTrkB.FL consumption after swimming, pointing to their degradation after their role as triggers of the pathway. Finally, decreased pTrkB.FL is a feature of slow muscles that occurs after swimming, thus representing a fast-to-slow adaptation (Table 1).

### 2.2. Serine-Threonine Kinases

**Figure 2:**
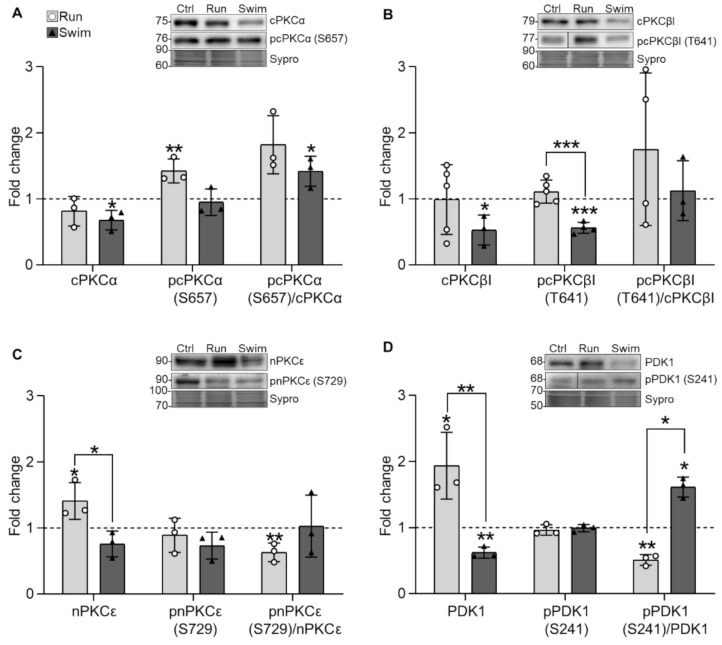
PKC isoforms and PDK1 expression changes in trained plantaris muscles of WT mice. (**A**) Running increases pcPKCα while swimming decreases cPKCα. (**B**) Swimming decreases both cPKCβI and pcPKCβI. (**C**) Running increases nPKCε. (**D**) Running increases PDK1 while swimming decreases it. Data are presented as mean ± SD. Each dot in the bars is the mean result of one animal. * *p* ≤ 0.05; ** *p* ≤ 0.01; *** *p* ≤ 0.001; (Kruskal–Wallis test and Dunn’s post hoc test).

Following NTFs and receptors changes, we investigated how exercise affects the ubiquitous cPKCα and the exclusive presynaptic cPKCβI and nPKCε isoforms in the plantaris muscles after the training protocols. The results reveal that running does not affect cPKCα total levels but increases pcPKCα (S657) while swimming decreases total cPKCα. Therefore, the pcPKCα/cPKCα ratio increases but only significantly after swimming (Figure 2A). Regarding cPKCβI, running neither affects total nor phosphorylated levels while swimming decreases both of them, similarly to the values observed in untrained WT soleus muscles (Table 1). Thus, after the two trainings, the pcPKCβI/cPKCβI (T621) ratio remains the same (Figure 2B). Finally, running increases nPKCε total levels and maintains pnPKCε (S729) while swimming affects neither of them. Consequently, the pnPKCε/nPKCε ratio is decreased after running and maintained after swimming (Figure 2C).

Furthermore, because PKC maturation and priming include a first phosphorylation step mediated by PDK1, we also analyzed it. Results show that running increases total PDK1 levels while swimming decreases it. Consequently, the pPDK1/PDK1 (S241) ratio decreases with run and increases with swim (Figure 2D).

Overall, these complex results show the increase of pcPKCα, nPKCε, and PDK1 protein levels after running and an important reduction of PDK1, cPKCα, and both cPKCβI and pcPKCβI after swimming. Interestingly, the exercise-induced values of cPKCβI after swimming are similar to the values observed in normal untrained slow soleus muscles rather than fast ones (Table 1).

**Figure 3:**
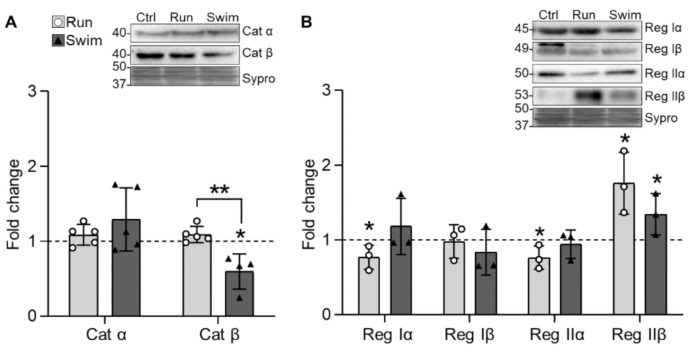
Catalytic and regulatory PKA subunits expression changes in trained plantaris muscles of WT mice. (**A**) Catalytic Catα never changes while Catβ decreases after swimming. (**B**) Regulatory RIα and RIIα decrease and RIIβ increases after running. Swimming increases RIIβ. Data are presented as mean ± SD. Each dot in the bars is the mean result of one animal. * *p* ≤ 0.05; ** *p* ≤ 0.01; (Kruskal–Wallis test and Dunn’s post hoc test).

We also analyzed the two catalytic PKA subunits (Cat α, Cat β) and the four regulatory subunits (Reg Iα, Reg Iβ, Reg IIα, and Reg IIβ) as the T138 phosphorylation of SNAP-25 (see Section 2.3.) depends on it to guarantee the correct size of the releasable vesicle pools [55]. Results do not show any change in Cat α but a decrease in Cat β after swimming (Figure 3A). In addition, running decreases Reg Iα and IIα and increases Reg IIβ, which is also increased by swimming (Figure 3B). Altogether, considering the stoichiometry of the subunits, it seems that PKA catalytic activity is only decreased after swimming. In particular, the strong and shared increase of the Reg IIβ, acquiring similar values to the ones in untrained soleus muscles (Table 1), may contribute to reducing PKA catalytic activity. This could be related with the optimization of the exocytotic process that occurs due to training habituation.

### 2.3. SNARE/SM Proteins

**Figure 4:**
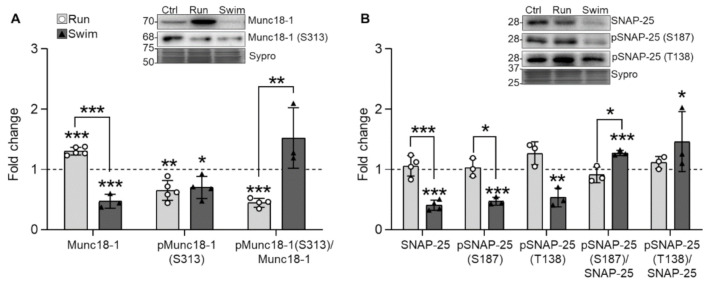
The SNARE/SM Munc18-1 and SNAP-25 expression changes in trained plantaris muscles of WT mice. (**A**) Running increases and swimming decreases Munc18-1 while both decrease pMunc18-1. (**B**) Swimming decreases SNAP-25 and its two phosphorylation residues. Data are presented as mean ± SD. Each dot in the bars is the mean result of one animal. * *p* ≤ 0.05; ** *p* ≤ 0.01; *** *p* ≤ 0.001; (Kruskal–Wallis test and Dunn’s post hoc test).

When looking at two of the serine-threonine kinases targets, the results show that running increases Munc18-1 and reduces pMunc18-1 (S313) while swimming reduces both Munc18-1 and pMunc18-1. Consequently, after running the pMunc18-1/Munc18-1 ratio is decreased while after swimming it is maintained (Figure 4A). Regarding the SNARE protein SNAP-25, it is unaffected by running but swimming significantly reduces its total levels and consequently, the phosphorylated ones (for both S187 -PKC dependent- and T138 -PKA dependent- residues). Accordingly, the ratios do not change after run but increase after swim (Figure 4B). Altogether, running modifies less the exocytotic proteins than swimming, probably due to its minor intensity. Interestingly, the reduction of the pMunc18-1 in running and the broad reduction of the Munc18-1 and SNAP-25, either phosphorylated or not, is also a feature of the slow muscles (Table 1).

### 2.4. Motor Neurons

Since exercise influences the NMJ, we aimed to investigate whether running and swimming-based training protocols could influence MNs. To do so, we detected choline acetyl-transferase (ChAT) to identify MN in lumbar spinal cord sections and analyze the soma size. On one-hand, ChAT-positive MNs with soma size up to 300 µm^2^ are defined as alpha MNs (αMNs). They are responsible of muscular contractions and can be classified, from larger to smaller, into fast-twitch fatigable (FF), fast-twitch fatigue-resistant (FR) and slow-twitch fatigue-resistant (S) subtypes, in accordance with the myocytes they innervate [56]. On the other hand, gamma MNs (γMNs) have smaller somas below 300 µm^2^, thinner axons, reduced conduction velocities [57], and regulate spindle sensitivity through the control of intrafusal myocytes contraction [56].

**Figure 5:**
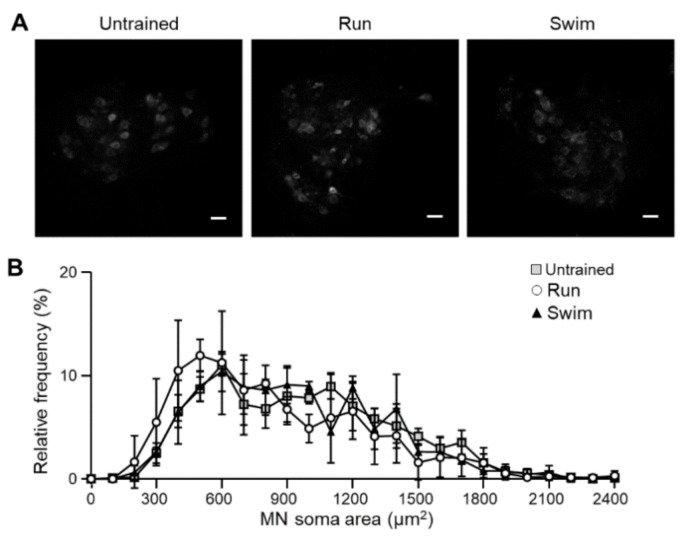
Motoneurons in the lumbar spinal cord of trained WT mice. (**A**) Immunohistochemistry images of the representative ChAT labelling in the tree groups of mice. No change in cell number is noticed. Scale bar: 50 μm. (**B**) MNs soma size are not modified by the trainings. Data are presented as mean ± SD. Kruskal–Wallis test and Dunn’s post hoc test did not reveal any significant change.

Results show that exercise does not modify the total number of MNs in the lumbar sections of the spinal cord. Furthermore, the proportion of small and slow MNs in relation with big and fast ones is maintained, for all MNs categories (Figure 5), indicating that muscular adaptations do affect neither in MN number nor soma area.

## 3. Discussion

Neurotrophic changes at the NMJ influence its stability and functionality [3,36,58,59]. The present results show that exercise strongly modifies, in the plantaris muscle, protein and phosphorylation levels of many molecules of the putative BDNF-NT4/TrkB-p75^NTR^/PKC-PKA/SNARE-SM pathway, which is essential to modulate NMJ maintenance and promote neurotransmission [3,50,51]. Table 1 summarizes the complex molecular changes after running and swimming training protocols and we discuss their meaning as they can explain a fast-to-slow fiber plastic molecular adaptation, according with previous findings [32].

### 3.1. Neurotrophic Factors and Receptors

Our results show that after a prolonged period of physical training (45 days) there is an increase of proBDNF, similar to the one that was found in proBDNF and BDNF mRNA after 120 min of electrical stimulation in muscle cells [38]. This suggests that the precursor protein would be increased due to mRNA increase. In fact, it has been reported that BDNF mRNA increases especially in slow muscles after physical training [9,10,60,61]. Therefore, despite the proBDNF levels not representing a fast-to-slow transition in the context of a sedentary soleus, as shown in Table 1, it coincides with the BDNF synthesis that would take place in a trained soleus.

On the other hand, acute (30 min) electrically-induced muscle contraction increases mBDNF without affecting mRNA in the mixed fiber type diaphragm [3], suggesting that short-term neuromuscular activity modulates the pathway by promoting translation and maturation while prolongated activity is able to modify gene transcription, as shown here. Furthermore, it has been described that mBDNF is secreted in response to exercise training of different duration and intensity in different skeletal muscles [38,62,63]. Here, we found that the great increase of proBDNF observed after running and swimming in the fast plantaris muscle is not accompanied by an increase of mBDNF. Indeed, mBDNF is differently regulated by each protocol, yet the mBDNF/proBDNF ratio is decreased in both protocols, pointing to a generalized accumulation of the precursor in trained animals that is differentially required. Thus, it seems that exercised myocytes synthesize a pool of proBDNF that can be cleaved into mBDNF on demand that should permit the binding of mBDNF to TrkB receptors to modulate synaptic activity when needed. This seems to be proportional to the intensity of the exercise as it is more pronounced in swimming than in running, coinciding with a fast-to-slow transition of mBDNF only after the swimming protocol. Alternatively, it cannot be discarded that the cleavage of proBDNF into mBDNF may be differentially reduced by the training activity in response to receptors availability.

NT4 is an activity-dependent neurotrophic signal for growth and remodeling NMJ. This is supported by the fact that exogenous NT4 induced sprouting of motor nerve terminals [64,65,66]. In accordance, NT4 mRNA decreased after neuromuscular transmission blockade and increased after electrical stimulation proportionally to intensity [67]. Furthermore, NT4 seems to be partly responsible for the effects of exercise and electrical stimulation on neuromuscular performance by avoiding damaged axons degeneration and inducing sprouting and reinnervation by healthy MNs and, thus, preventing muscular denervation [36,67]. Thus, NT4 could positively regulate the molecular adaptations due to the training described here. In fact, NT4 levels going beyond mBDNF ones resemble the soleus values (Table 1).

In addition, NT4 requires more time than mBDNF to activate TrkB endocytosis and stop downstream signaling [68]. Thus, the accumulation of NT4 seems to be in accordance with the molecular fast-to-slow transition that plantaris undergoes, especially after swimming, which seems to slow down the downstream signaling. Altogether, these changes in the NTFs modulate the fast-to-slow transition induced by running and swimming endurance training and the changes in the downstream pathway support this interpretation.

During the development and after muscle stimulation, proBDNF promotes synaptic plasticity and refinement through p75^NTR^ [39,40,69]. Accordingly, it seems that exercise, like in development, would promote p75^NTR^-mediated plasticity mechanisms to refine and optimize mature adult NMJ connections. However, despite proBDNF increases after both protocols, p75^NTR^ does not behave symmetrically. This coincides with the fact that swimming muscles, where p75^NTR^ is downregulated, attain a more complete fast-to-slow molecular adaptation than running muscles, which coincides with a faster morphological and physiological fast-to-slow transition [70]. Thus, in running, the p75^NTR^ accumulation may represent a signaling that is going through the molecular adaptation while in swimming p75^NTR^ is less expressed as its adaptative mission may have been already achieved. These data add new evidence that p75^NTR^ has a role on synapse refinement also in adult animals [71].

Heterodimers of TrkB.FL with the truncated isoforms inhibit TrkB.FL trans-autophosphorylation, reducing BDNF signaling [44,72,73,74,75]. In the skeletal muscle, TrkB.T1 is the main isoform and acts in a dominant negative fashion to decrease TrkB.FL signaling [44,45,46]. Indeed, Dorsey et al., (2012) [73] demonstrated that the deletion of TrkB.T1 increases neurotrophic factor-dependent activation of downstream signaling targets and increases contraction. Accordingly, when muscle contraction is electrically promoted, TrkB.T1 is downregulated in favor of TrkB.FL, which is increased. In this situation, the downstream signaling through pTrkB.FL (Y816) is more active without altering p75^NTR^ levels [3,76].

Here, results show that after a long period of run or swim training, the TrkB isoforms change differently and so the ratios do. Thus, although TrkB.FL increases in both training protocols, pTrkB.FL only increases moderately after running and is reduced after swimming. The abundance of TrkB.T1 in running probably may downregulate the formation of pTrkB.FL, decreasing the downstream signaling. On the other hand, despite TrkB.T1 being unaltered after swimming, pTrkB.FL is strongly decreased. It could be that pTrkB.FL is being consumed due to utilization, but probably TrkB.FL phosphorylation is restricted as a result of the fast-to-slow transition, as the generalized decrease in the downstream pathway shows (see below). Overall, results suggest a downregulation of the TrkB pathway in accordance with the phenotypic fast-to-slow transition that the system undergoes, directly opposite to the adaptation to acute electrical stimulations [3,76]. Furthermore, pTrkB.FL is less abundant in slow muscles, like the soleus, in comparison with fast plantaris ones (Table 1) [32]. In accordance with that, swimming, which is more intense and achieves the fast-to-slow transition in a shorter time than running [70], seems to optimize the BDNF/TrkB signaling faster to adapt it to the new situation. Indeed, similar results have been found in the hippocampus of treadmill-trained transgenic mice [77,78].

### 3.2. Serine Threonine Kinases and Their Targets

#### 3.2.1. PDK1 and PKC

Phosphorylated Y816 TrkB.FL residue activates the phospholipase Cγ (PLCγ) pathway, generating inositol-1,4,5-triphosphate (IP3), and diacylglycerol (DAG). This results in calcium release from intracellular stores that activates PKCs [79]. At the NMJ, PKCs are then translocated to the membrane for further activation and phosphorylation of targets [3]. As each training protocol differently modifies the first step of the BDNF-NT4/TrkB- p75^NTR^ interaction, we analyze the downstream proteins in the chain, PDK1, and PKC isoforms.

PDK1 directly regulates the priming of the presynaptic PKC isoforms nPKCε and cPKCβI and, consequently, their activity [47]. Furthermore, they are activated through BDNF/TrkB after acute electrically-induced muscle contraction to modulate neuromuscular transmission [3,48,80]. The present results show that each training protocol differently modifies PDK1 activity in accordance with the previous step in the pathway. Thus, after running there is an increase of PDK1 following the moderate increase of pTrkB.FL activity while, after swimming, the decrease of pTrkB.Fl results in less total PDK1. However, previous results reported that presynaptic PDK1 is constitutively active [47], which is in accordance with unaltered pPDK1 after both trainings.

Consequently, after running, cPKCβI maintains its levels and activity while pcPKCα and nPKCε increase (in these last cases as in slow muscles) [32]. In accordance with previous results that reported that Munc18-1 synthesis depends on nPKCε [51], here, their levels are modulated in the same direction. However, Munc18-1 phosphorylation is differently modulated as it is regulated by both cPKCβI and nPKCε at the NMJ [51]. Therefore, it seems that the regulation of the balance between cPKCβI and nPKCε that modulates their phosphorylating activity is decreased in relation with Munc18-1. This would link the decrease of TrkB.FL activity due to TrkB.T1 inhibition that directly reduces cPKCβI activity with the decrease of pMunc18-1. As cPKCα is ubiquitous while cPKCβI and nPKCε are exclusively presynaptic [49] and their implication over Munc18-1 and SNAP-25 is known [50,51], the modulation of the last two has been exclusively interpreted in key presynaptic components. Thus, cPKCα activity has been interpreted to globally affect the fast-to-slow transition (see below) but not precisely the phosphorylation of Munc18-1 and SNAP-25.

On the other hand, SNAP-25 synthesis and phosphorylation at S187 are independent of cPKCβI and exclusively depend on nPKCε [50]. Because nPKCε is influenced by muscarinic receptors and PKA, its activity seems to be maintained despite of TrkB signaling reduction, thus allowing the maintenance of SNAP-25 and pSNAP-25 (S187) levels. In this case, this is in concordance with electrical stimulation-induced muscle contraction, which maintains the pool of total and phosphorylated SNAP-25 [50].

Swimming reduces Munc18-1 and SNAP-25 total levels either due to consumption after usage or lack of synthesis but always in the direction of a fast-to-slow transformation (Table 1). Interestingly, they coincide with decreased cPKCβI and TrkB.FL levels and activity, which are also features of the slow muscles when compared with fast ones (Table 1) [32]. This clearly indicates a fast-to-slow transition of the exocytosis components after swimming, revealing a mechanism of neurotransmission adapted to resist prolonged stimulation. This effect is clearly seen in swimming, probably because it is more intense than running and exacerbates molecule utilization and following degradation and faster adaptation of the entire signaling pathway into a slow-type NMJ [81]. However, running induces a powerful enough change in pTrkB.FL that triggers the fast-to-slow adaptation although more slowly.

It has been reported that in skeletal muscles, cPKCs are activated by fast, but not slow, patterns of activity [82] and do not seem to be affected by exercise in humans [83]. Furthermore, in avian models, PKC activity is higher in fast than in slow muscles. Indeed, an elevation of cPKCα (and nPKCθ) resulted in a slow-to-fast conversion [84,85,86]. Thus, it seems that cPKCs might sense fast activity patterns to modulate MyHC isoforms expression in correspondence [82]. These data reinforce our findings of decreased cPKCα and cPKCβI activity, especially after swimming training, in the context of a metabolic fast-to slow transition.

Additionally, although nPKCs lack the calcium binding domain, the Ca^2+^ influx induces DAG production that does translocate and activate them. Thus, nPKCs could also work as a pattern-specific signal decoder of nerve-evoked activity in muscle. Indeed, Protein kinase D1 (PKD1), a downstream target of nPKCθ and nPKCε, is especially abundant in slow-type muscles [87,88]. Accordingly, the fast-to-slow transition induced by training does not decrease nPKCε activity. Therefore, nPKCs and cPKCs might play opposite roles in the fast-to-slow transition that converge to promote it. In particular, the activation of cPKC promotes the activity of fast genes and therefore their inhibition (or reduction of their activity) would promote the fast-to-slow transition while the activity of nPKC would directly promote the expression of slow genes that also promote the fast-to-slow transition [82].

#### 3.2.2. PKA

To investigate PKA function in the presynaptic component of the NMJ, the phosphorylation of its exclusively presynaptic target SNAP-25 has been studied, in accordance with its implication in acetylcholine release [50]. This ensures that the present PKA activity analysis mainly concerns the presynaptic component, despite of being active in the postsynaptic or glial components. Regarding the PKA subunits, we found a moderate change in the stoichiometry of the regulatory subunits versus the catalytic ones after running that seems to not change PKA activity as the PKA substrate pSNAP-25 (T138) is unaffected. On the other hand, swimming decreases PKA function through a reduction of the Cβ and a large increase of the RIIβ. Accordingly, pSNAP-25 (T138) also decreases, which is representative of a fast-to-slow transition [32] and coincides with the optimization of the signaling after applying the endurance swimming protocol. Furthermore, as PKA is also related with protein synthesis, the decrease of Cβ after swimming would support total Munc18-1 and SNAP-25 decrease, which is similar to the values found in the soleus (Table 1) [32].

Finally, PKA phosphorylation of SNAP-25 in the T138 residue is directly related with the control of the size of the releasable vesicle pools [55,89]. MEPPs frequency and quantal content are minor in slow muscles than in fast ones [90]. However, long periods of exercise increase them together with the safety factor accompanied by a gain of resistance in the myocytes [15,18,91,92]. Thus, decreased SNAP-25 and pSNAP-25 (T138) not only represent the fast-to-slow transition [32] but also show the molecular relation with the functional adaptation of the NMJ to exercise. This parameter is only achieved by swimming, pointing to the fact that the fast-to-slow transition is proportional to the intensity of the training. Moreover, these results at the nerve terminal are coincident with the functional coupling between PKA and calcineurin to modulate the fast-to-slow transition in the muscular fibers [93]. In brief, calcineurin dephosphorylates to activate NFAT and allows its translocation to the nucleus where it upregulates slow muscle genes [94] and downregulate fast ones in an activity-dependent manner [95]. Consequently, calcineurin enhances the transcription of slow specific genes [96] and decreased PKA activity facilitates it.

### 3.3. BDNF/TrkB Impact over the Morphology of the Neuromuscular System

The present results confirm that exercise protocols are not harmful for healthy mice MNs and that these cells do not change their soma surface despite of the molecular adaptations in the muscles they innervate. MN distribution follows an approximate Gaussian distribution, being the 500–600 μm^2^ range the more abundant ones for the three groups. There is no difference in neither the number nor size of MN somas despite of the molecular adaptations reported at the NMJ. These results are in concordance with previous ones where no changes in soma volume due to exercise were found [97,98,99]. On the contrary, changes in MN dendritic size and number [100] and functional properties from “fast” to “slow” together with changes in myocytes were found [101].

Physical exercise induces reversible changes that mediate tissue adaptability [102] to respond to new situations without affecting the general character of cells. Thus, endurance protocols adapt gene expression towards a slower metabolic profile, indicating that MNs work differently as they undergo metabolic adaptations, including increased protein synthesis [103,104], transport [105,106,107,108], and secretion of trophic factors that influence transcription of subsynaptic myonuclei but they still maintain their own identity. In parallel, myocytes maintain the expression of myosin heavy chains but adapt their metabolism to the new requirements [70] and increase myokine release [38]. In brief, as a result of exercise, Type II fibers follow a fast-to-slow transition in which they adopt a more oxidative metabolism due to changes in gene expression [109]. This increases resistance to fatigue thanks to improved mitochondrial function [110,111,112,113] and is principally mediated by peroxisome proliferator-activated receptor gamma coactivator 1-alpha (PGC-1α) expression [114,115,116]. In particular, exercise-induced BDNF paracrinally optimizes neurotransmission at the nerve terminal [3,48,50,51] while autocrinally increasing fat oxidation at the myocytes through AMPK activation [38], which potentiates PGC-1α action by phosphorylating it [117]. Altogether, exercise could induce, at least, two parallel and interconnected adaptations, one in the presynaptic and one in the postsynaptic components, that contribute to the fast-to-slow transition and that are mediated by BDNF and that go through NMJ plastic capacity. With this, the adaptation of the entire motor units is guaranteed. Interestingly, SNAP-25 is selectively transported in higher quantities in axons of trained MNs to increase transmission efficacy [108].

Accordingly, exercise modifies NMJs morphology and functionality patterns [15,16,17,18,19,20,21,22,23,24,25,26,27,118,119] to enhance synaptic efficacy and neurotransmitter release [15,120]. Despite of divergent results, it seems that the presynaptic component grows and nerve terminals are refined after endurance [18,121] in spite of the muscle fiber type [20,122]. Furthermore, running increases the subcellular structures related with the neurotransmission process, including the number of synaptic vesicles, the length and number of active zones, the postsynaptic folds number and the surface occupied by AChRs in the fast EDL muscle [17], which can be considered an attempt to increase the efficacy of the neurotransmission machinery. On the other hand, the postsynaptic component was only modified by endurance in fast NMJs, revealing an increased endplate perimeter [123]. However, acetylcholinesterase increases in in the synaptic cleft [124] and acetylcholine receptors in the postsynaptic component [125]. Accordingly, large and well-organized active zones in the fast muscles could produce phasic bursts of high activity in the synapse leading to high intracellular calcium levels in the presynaptic terminal resulting in a metabolic adaptation towards the slow phenotype.

Neurotrophic factors as NT-4 and BDNF could mediate the effect of exercise over the NMJ (reviewed in Nishimune et al., 2014) [21]. In particular, BDNF prevents muscle denervation and atrophy [126,127,128,129] and NT-4 promotes nerve terminal sprouting [67] and enhances neurotransmission [36] through TrkB signaling, which is essential to maintain synaptic function and structural integrity at NMJs in the adult mice [3,33,34,35,37]. Consequently, TrkB signaling disruption harm NMJ morphology [36,58,130,131,132]. Thus, muscle-derived BDNF retrogradely regulates neurotransmission at the presynaptic nerve terminal to make it as efficient as possible. Moreover, changes induced by exercise seem to be an activity-dependent plasticity mechanism modulating the NMJ performance mediated by PGC-1α [118] that increases BDNF production after exercise [63,133], thus closing a bidirectional feedback loop in which BDNF is a mediator for the fast-to-slow transition in skeletal muscles [132].

Thus, endurance protocols adapt the expression of genes in MNs and muscles towards a slower metabolic profile, indicating that both work differently after a training period despite that they still maintain their own identity. Similarly, under different training conditions, myocytes maintain the expression of myosin heavy chains but adapt their metabolism to the new requirements [70].

## 4. Materials and Methods

### 4.1. Animals

Wild-type (WT) B6SJLF1/J male mice (Janvier, le Genest, France) until 115 days of age were kept in the animal facility under standard conditions: constant temperature (22 ± 2 °C), relative humidity (50 ± 10%), and a 12-h light/dark schedule and unrestricted access to food and water. Animal handling and experimentation were performed in line with approved Institutional Animal Care and Use Committee protocols at the University of Paris Descartes (CEEA 34, agreement number B75-06-07, accepted the 29 September 2014, for 5 years) and followed the national authority (Ministere de la Recherche et de la Technologie, Paris, France) guidelines for the detention, use and the ethical treatment of laboratory animals based on European Union Directive 2010/63/EU.

### 4.2. Training Protocol

Mice were trained from 70 days of age (young adults), 30 min a day, five days a week for six weeks, until 115 days of age as previously described [70]. Five mice were submitted to a moderate running-based training on a speed-regulated treadmill (max. 13 m min^−1^) (Run) which is a low amplitude and frequency exercise that preferentially activates small MNs belonging to slow motor units and five mice were submitted to a high-frequency and amplitude swimming-based training in an adjustable-flow swimming pool (max. 5 L min^−1^) (Swim) which is a high amplitude and frequency exercise that also activates large MNs, belonging to fast motor units. These groups were compared with five untrained mice that only displayed an exploratory activity during the training time of the first and second groups. Sample size was calculated using previously stablished criteria [134,135] to optimize the number of animals used.

### 4.3. Western Blotting

Four hours after the training was complete (P115), the animals were euthanized and plantaris muscles were dissected and immediately frozen in liquid nitrogen. Plantaris are fast-twitching extensors of the ankle which makes them a good model to study skeletal muscle adaptation to endurance training, either running or swimming. Plantaris muscles have been chosen because fast muscles are more susceptible to training effect [30,31] and the putative fast-to-slow molecular transition is more evident. Particularly, plantaris motor units are almost 100% fast, which discards the possibility of slow ones interfering in the results. Muscles from both hind limbs of the same animal were processed and analyzed together by Western blot.

Western blotting procedure was performed as previously described [32]. Muscles were dissected and frozen in liquid nitrogen and homogenized using a manual homogenizer in ice-cold lysis buffer (in mM: NaCl 150, Tris-HCl (pH 7.4) 50, EDTA 1, NaF 50, PMSF 1, sodium orthovanadate 1; NP-40 1%, Triton X-100 0.1%, and protease inhibitor cocktail 1% (Sigma-Aldrich, Saint Louis, MO, USA). Protein lysates were obtained collecting supernatants after removing insoluble materials by centrifugation at 4 °C and aliquots were stored at −80 °C. Protein concentrations were determined by DC protein assay (Bio-Rad, Hercules, CA, USA).

Protein samples of 30 µg were separated by 8% or 12% SDS-polyacrylamide electrophoresis and electro-transferred to a polyvinylidene difluoride (PVDF) membrane (Hybond™-P; Amersham, GE Healthcare, Marlborough, MA, USA) using Trans-Blot Turbo Transfer System (Bio-Rad, Hercules, CA). For immunodetection, membranes were blocked with TBST containing 5% (*w*/*v*) phosphoblocker or bovine serum albumin (BSA) for phosphorylated proteins and non-fat dry milk for non-phosphorylated proteins for an hour. Then, membranes were incubated in primary antibody (Table 2) overnight and with a corresponding secondary antibody horseradish peroxidase-conjugated (Table 2) for one hour. Primary antibodies were omitted from some samples during the procedure as controls, and they never revealed bands of the appropriate molecular weight. Furthermore, their specificity has been proved in previous publications [3,47,50,51,52].

Membranes were revealed with Bio-Rad ECL kid on the ChemiDoc XRS+ machine (Bio-Rad, Hercules, CA, USA). The bands optical density was normalized in relation to (1) the background values and to (2) the total protein transferred on PVDF membranes, measured by total protein analysis (Sypro Ruby protein blot stain, Bio-Rad, Hercules, CA, USA [136]). The relative variations between samples were calculated from the same membrane image. Data was taken from densitometry measurements made in at least three separate western blots. Specific phosphorylation proportion was determined as the ratio of phosphorylated protein over total protein content. Ratios are represented in the histograms to facilitate the relation between the interpretation in results and discussion and the individual phosphorylated and total protein values.

### 4.4. Immunohistochemistry

The spinal cord of P115 mice was also dissected after the animals were anaesthetized by intraperitoneal injection of 1% pentobarbital solution (6 µL·g^-1^) and transcardially perfused with 20 mL of buffered saline then spinal cord were dissected and fixed in 4% paraformaldehyde overnight. The L1–L5 lumbar region of the spinal cord was sectioned with a vibrating blade microtome (VT-1000S, Leica Microsystems SAS, Nanterre, France) at 50 μm thickness. One out of every six sections was processed for immunostaining on free-floating sections (an average of seven sections per animal were studied). The immunohistochemical analysis was based on detection of choline acetyltransferase (ChAT) to stain motoneurons. Primary and secondary antibodies conjugated to Alexa fluor used are specified in Table 2. The staining specificity was checked by performing the incubation in the absence of the primary antibody. Sections were mounted with Vectashield mounting medium (Vector Laboratories, Burlingame, CA, USA) and images were collected using a CMOS camera (ORCA Flash 2.8, Hamamatsu Photonics France, Massy, France) mounted on a Zeiss Axio Observer microscope (Z1, Carl Zeiss SAS, Le Pecq, France) using ZEN 2012 software (Carl Zeiss SAS, Le Pecq, France). At least 100 MNs for each animal were counted.

### 4.5. Statistical Analysis

All values are represented as mean ± standard deviation (SD) within each group. Also, each dot represents the value of one animal to visualize their distribution. Statistical significance of the differences between the experimental groups was evaluated under a non-parametric Kruskal–Wallis test followed by Dunn’s post hoc test (GraphPad Prism software, San Diego, CA, USA). The criterion for statistical significance was: * *p* < 0.05, ** *p* < 0.01, and *** *p* < 0.001.

## 5. Conclusions

Physical exercise is a key modulator of the plasticity of muscles and nerves. Its results depend on the type, intensity, and duration of the induced contractions to trigger different intracellular signaling pathways. Activity dependent plastic modulation of the neurotransmission related structures in the normal adult occur in response to functional demands. Thus, the NMJ components change to adapt to new situations. Presynaptic nerve terminals are optimized regarding to the organelle machinery, branching complexity, and membrane densifications and active zones to sustain synaptic transmission. Moreover, postsynaptic membrane densification and fold crests adjust themselves and nAChR increases. Additionally, myocytes adapt their gene expression to a slower phenotype.

The main result of the present study indicates that the change in the protein level of most BDNF/TrkB pathway and downstream neurotransmitter release related molecules in the trained fast plantaris resembles to levels in untrained slow muscles. Interestingly, slow muscles have lower quantal content and spontaneous quantal release frequency than fast muscles, due to their functional properties [90]. However, similar electrophysiological studies in trained muscles indicated that endurance training results in quantal content and safety margin increase. Here, we relate the molecular adaptations that fast, trained plantaris undergo to adapt to the new functionality requirements and show that a slower phenotype does not require a full molecular transformation.

Our results demonstrate that the BDNF/TrkB pathway, one of the most implicated in NMJ preservation, is precisely modulated by exercise, depending on the nature and intensity of the training, which permits to understand the benefits that physical exercise has over our health. Altogether, it seems that both training protocols studied optimize the BDNF/TrkB signaling pathway as, through the training, an amount of work is achieved to adapt the muscle to optimize its resources, which results in a transition to a slow way of working. This seems to have the objective to acquire a prolongated resistance and more stability to resist effectively prolonged trains of stimulation or activity. The molecular fast-to-slow transition induced by endurance exercise observed in the BDNF/TrkB signaling at the plantaris skeletal muscle occurs with some independence of the soma, where no changes in size occurs, thus, pointing to the autonomous capacity of the nerve terminal to respond to activity changes. However, further analysis of changes in MN soma gene transcription would be of interest, despite the morphology not reflecting them.

## Figures and Tables

**Table 1:** Summary of exercise-induced molecular adaptations. (**A**) Fast-to-slow transition following either running or swimming training protocols. In the table, blue-shaded cells highlight the values that after the training protocol are statistically similar to those in sedentary mice soleus muscles. Yellow highlights the values that after the training protocol are opposite to those in sedentary mice soleus, being statistically different from both untrained soleus and plantaris. Notes: Lighter colors correspond to these changes induced by trainings despite that no significative differences were found between untrained plantaris and soleus muscles. To facilitate data analysis, the first column corresponds to the values of soleus muscles in relation with plantaris (both untrained) that were published for first time in [32]. Statistical significance of the differences between the experimental groups was evaluated under a non-parametric Kruskal–Wallis test followed by Dunn’s post hoc test. The criterion for statistical significance against WT plantaris was as follows: * *p* < 0.05, ** *p* < 0.01, and *** *p* < 0.001 (precise *p*. value is provided). (**B**) Summary of the results of this work. Representation of the molecular changes in the BDNF/TrkB signaling pathways after run (**left**) and swim (**right**) training protocols. The figure shows that molecular changes are more abundant and mainly in the fast-to-slow direction after the swimming protocol, due to the intensity-dependent effect.

A		SOL WT					PLA WT Run					PLA WT Swim				
NTFs and receptors	proBDNF	1.05	±	0.31	0.921		2.65	±	0.89	0.008	**	2.17	±	0.93	0.045	*
	mBDNF	0.57	±	0.27	0.021	*	0.95	±	0.29	0.736		0.45	±	0.18	0.002	**
	NT4	1.89	±	0.03	0.000	***	1.81	±	0.10	0.000	***	1.51	±	0.14	0.000	***
	p75^NTR^	1.11	±	0.03	0.393		1.68	±	0.24	0.001	**	0.79	±	0.10	0.006	**
	TrkB.T1	0.92	±	0.06	0.619		2.45	±	0.14	0.000	***	0.92	±	0.14	0.305	
	TrkB.FL	0.63	±	0.04	0.012	**	1.95	±	0.62	0.022	*	1.84	±	0.54	0.021	*
	pTrkB.FL (Y816)	0.19	±	0.01	0.000	***	1.23	±	0.14	0.014	*	0.40	±	0.16	0.000	***
Serine-threonine kinases	PDK1	1.35	±	0.09	0.089		1.94	±	0.51	0.033	*	0.62	±	0.09	0.002	**
	pPDK1 (S241)	0.99	±	0.26	0.998		0.96	±	0.08	0.452		0.99	±	0.05	0.824	
	cPKCα	1.19	±	0.01	0.258		0.81	±	0.22	0.222		0.68	±	0.15	0.020	*
	pcPKCα (S657)	0.61	±	0.03	0.014	**	1.42	±	0.18	0.008	**	0.95	±	0.20	0.688	
	cPKCβI	0.15	±	0.11	0.000	***	0.99	±	0.53	0.976		0.53	±	0.23	0.023	*
	pcPKCβI (T621)	0.51	±	0.22	0.000	***	1.11	±	0.18	0.328		0.56	±	0.08	0.000	***
	nPKCε	1.65	±	0.32	0.000	***	1.41	±	0.28	0.017	*	0.76	±	0.20	0.099	
	pnPKCε (S729)	0.77	±	0.06	0.140		0.89	±	0.26	0.508		0.73	±	0.20	0.085	
	PKA Cα	2.04	±	0.07	0.000	***	1.09	±	0.14	0.270		1.29	±	0.42	0.215	
	PKA Cβ	1.13	±	0.43	0.648		1.09	±	0.11	0.147		0.60	±	0.24	0.014	*
	PKA RIα	2.4	±	0.56	0.000	***	0.77	±	0.16	0.032	*	1.18	±	0.37	0.364	
	PKA RIβ	1.15	±	0.01	0.776		0.98	±	0.22	0.846		0.83	±	0.31	0.312	
	PKA RIIα	0.79	±	0.09	0.583		0.76	±	0.16	0.027	*	0.94	±	0.19	0.553	
	PKA RIIβ	1.98	±	0.07	0.006	**	1.76	±	0.41	0.013	*	1.34	±	0.28	0.049	*
Exocytosis machinery	Munc18-1	0.75	±	0.12	0.081		1.30	±	0.06	0.000	***	0.47	±	0.12	0.000	***
	pMunc18-1 (S313)	0.54	±	0.11	0.000	***	0.65	±	0.17	0.004	**	0.70	±	0.18	0.017	*
	SNAP-25	0.75	±	0.17	0.050		1.06	±	0.17	0.515		0.40	±	0.08	0.000	***
	pSNAP-25 (S187)	2.31	±	0.06	0.000	***	1.03	±	0.15	0.700		0.47	±	0.07	0.000	***
	pSNAP-25 (T138)	0.35	±	0.21	0.000	***	1.27	±	0.19	0.158		0.53	±	0.16	0.002	**
**B**	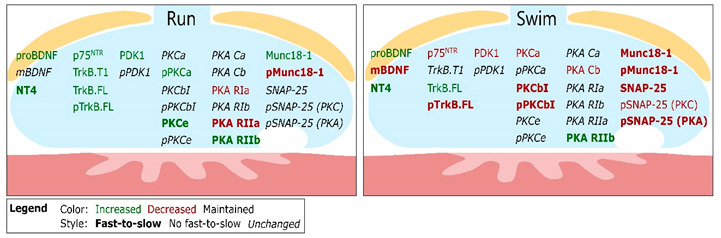															

**Table 2:** List of primary and secondary antibodies used.

Target	Source	Reference	Dilution	Target	Source	Reference	Dilution
BDNF	Rb pAb	Sc-20981	1/500	PKA Cα	Rb pAb	Sc-903	1/1000
NT4	Rb pAb	Sc-545	1/500	PKA Cβ	Rb pAb	Sc-904	1/1000
p75^NTR^	Rb pAb	07-476	1/800	PKA RIα	Ms mAb	Sc-136231	1/1000
TrkB	Ms mAb	Sc-377218	1/1000	PKA RIβ	Rb pAb	Sc-907	1/1000
pTrkB (Y816)	Rb pAb	ABN1381	1/1000	PKA RIIα	Rb pAb	Sc-909	1/1000
PDK1	Ms mAb	Sc-17765	1/1000	PKA RIIβ	Ms mAb	Sc-376778	1/1000
pDPK1 (S241)	Rb pAb	#3061	1/1000	Munc18-1	Rb mAb	13414	1/1000
cPKCα	Rb pAb	Sc-208	1/800	pMunc18-1 (S313)	Rb pAb	Ab138687	1/1000
pcPKCα (S657)	Rb pAb	06-822	1/1000	SNAP-25	Rb mAb	#5309	1/1000
cPKCβI	Rb pAb	Sc-209	1/1000	pSNAP-25 (S187)	Rb pAb	Ab169871	1/1000
pcPKCβI (T641)	Rb pAb	Ab75657	1/1000	pSNAP-25 (T138)	Rb pAb	Orb163730	1/1000
nPKCε	Rb pAb	Sc-214	1/1000	HRP-conjugated	Dk a-Rb pAb	711-035-152	1/10.000
pnPKCε (S729)	Rb pAb	Sc-12355	1/1000	HRP-conjugated	Rb a-Ms pAb	A9044	1/10.000
ChAT	Gt pAb	AB144P	1/400	Cy3	Dk a-Gt pAb	705-165-003	1/400

## Data Availability

The data presented in this study are available on request from the corresponding author.
